# Targeting BCR-ABL1-positive leukaemias: a review article

**DOI:** 10.1017/pcm.2023.9

**Published:** 2023-03-06

**Authors:** Steven Leak, Gillian A. Horne, Mhairi Copland

**Affiliations:** 1 Beatson West of Scotland Cancer Centre, NHS Greater Glasgow and Clyde, Glasgow, UK; 2 Paul O’Gorman Leukaemia Research Centre, School of Cancer Sciences, University of Glasgow, Glasgow, UK

**Keywords:** Chronic myeloid leukaemia, tyrosine kinase inhibitor, leukaemia stem cell, BCR::ABL1

## Abstract

Treatment and understanding of BCR::ABL1-positive leukaemias is a precision medicine success story. Our appreciation of the *BCR::ABL1* gene and resulting BCR::ABL1 oncoprotein in chronic myeloid leukaemia (CML) and Philadelphia chromosome-positive (Ph+) acute leukaemias, has led to treatment advances associated with exceptional improvements in patient outcomes with normal life expectancy for many patients with chronic phase (CP-)CML. However, despite these major therapeutic advances, the management of Ph+ leukaemias remains complex, with development of specific resistance mutations on treatment, as well as the need for lifelong therapy in most patients due to the persistence of CML stem cells despite prolonged tyrosine kinase inhibitors (TKIs) treatment. BCR::ABL1-specific TKIs are associated with chronic toxicities affecting quality-of-life in many patients but can also result in more serious pulmonary and cardiovascular complications. Dose optimisation is increasingly being used to manage side effects and maintain molecular response in CML patients. Here, we review the development of BCR::ABL1-specific TKIs from the discovery of imatinib in 1996 to the more recent second- and third-generation TKIs and emerging specifically targeting the ABL myristoyl pocket (STAMP) inhibitors. We will also evaluate the current evidence for treatment of BCR::ABL1-positive leukaemias, including TKI discontinuation in optimally responding CP-CML patients.

## Impact statement

Leukaemia describes a mixed group of cancers affecting blood cell development. Its management has changed drastically over the past 40 years and with this, we have seen significant improvements in patient outcomes. These advances have come about through continued research into the underpinning mechanisms driving leukaemia development. In addition to better treatments for leukaemia, our understanding of the genes that cause leukaemia has improved. This review focuses on the blood cancer-causing gene *BCR::ABL1* which results from an abnormal join between two chromosomes, 9 and 22. The *BCR::ABL1* gene most commonly leads to a blood cancer called chronic myeloid leukaemia (CML), but can also cause an acute leukaemia, more commonly acute lymphoblastic leukaemia and rarely, acute myeloid leukaemia. Our knowledge of how the *BCR::ABL1* gene and resulting protein drive cancer cell progression and survival through complex processes within the cell which promote cell growth and switch off normal cell death pathways. At the same time, targeted treatments have been developed which directly stop BCR::ABL1 from affecting cell growth and survival, replacing traditional, more toxic chemotherapy drugs which have a blanket effect on all developing cells, both healthy and cancer cells. These new drugs are called tyrosine kinase inhibitors or “TKIs”; the first of which was designed nearly 25 years ago and is now called imatinib (trade name “Gleevec” or “Glivec”). TKIs have led to much improved survival for patients, especially with CML, but also reduced toxicity and improved quality of life. Indeed, patients with the most benign phase of CML, termed chronic phase, if responding to these targeted drugs, can expect normal life expectancy. This paper will discuss the advances made in BCR::ABL1-positive leukaemias, and discuss ongoing issues with their use and highlight where research must now focus to continue to improve outcomes for patients through precision medicine.

## Background

First described in the 19th century, the term “leukaemia” describes a heterogenous group of conditions affecting the various levels of haematopoietic cell development (Bennett, 1845). More recently, our understanding of leukaemias continues to develop, and with advances in genetic and epigenetic mechanisms, as well as diagnostics, our appreciation of the underpinning pathogenesis leading to these malignant clones has brought with it targeted treatment advances.

Chronic myeloid leukaemia (CML) represents an excellent model where understanding of disease pathogenesis has been translated into clinical practice, and continues to improve outcomes for patients. This article will focus on the development of tyrosine kinase inhibitors (TKIs) in CML, as well as the emerging specifically targeting the ABL myristoyl pocket (STAMP) inhibitors. We will go on to discuss the side-effect profiles relating to both “on-target” and “off-target” mechanisms via kinase inhibition, and briefly discuss their emerging roles in Philadelphia chromosome-positive acute lymphoblastic leukaemia (Ph+ ALL) and Ph+ acute myeloid leukaemia (Ph+ AML).

## Chronic myeloid leukaemia and the discovery of *BCR::ABL1*

CML accounts for 15–20% of all adult leukaemias with an incidence of around 1 in 100,000. Median presentation is between 40 and 60 years. Classically, patients present in chronic phase (CP) disease with high white cell count and splenomegaly, and without medical intervention, will progress to blast phase (BP), which behaves like an acute leukaemia with dismal outcomes. CML was previously described as a triphasic disease, but recent WHO guidelines have removed accelerated phase as a disease entity, in keeping with recent scientific advancement (Khoury et al., 2022).

Historically, initial treatment involved standard cytotoxic chemotherapy, including busulphan and hydroxycarbamide. Definitions of treatment response in CML are described in Table 1. Although a haematological response was obtained with this approach, no cytogenetic response could be achieved as BCR::ABL1 was not being targeted specifically (Hehlmann et al., 1993). Interferon alpha was introduced as a standard-of-care in the mid-1980s, and demonstrated a significant improvement when compared to preceding therapies, with a 15% higher 5-year survival (Talpaz et al., 1986). Cytogenetic response and overall survival were improved when combined with systemic chemotherapy, such as cytarabine (Beck et al., 2001). Within the 1980s, it also became clear that allogeneic haematopoietic stem cell transplantation (alloHSCT) could result in long-term disease-free survival and probably cure of the disease for selected patients, though not without significant risk of morbidity and mortality (Fefer et al., 1979; Clift et al., 1982).

**Table 1.** Definitions of response in CML, international assessment scale

Response	Definition
Complete haematological response (CHR)	White cell count (WCC) and platelet count have returned to normal and no immature cells seen in peripheral blood by morphology. Resolution of splenomegaly.
Major cytogenetic response (MCyR)	≤35% of cells in peripheral blood have the *BCR::ABL1* transcript or are Ph+ (minimum of 20 cells examined for conventional cytogenetics)
Partial cytogenetic response (PCyR)	1–35% of cells in peripheral blood have the *BCR::ABL1* transcript or are Ph+ (minimum of 20 cells examined for conventional cytogenetics)
Complete cytogenetic response (CCyR)	≤1% *BCR::ABL1* transcripts or are Ph+ (minimum of 20 cells examined for conventional cytogenetics)
Major molecular response (MMR; MR3)	≤0.1% *BCR::ABL1* transcripts against 10,000 *ABL* reference gene transcripts/24,000 *GUSB* reference gene transcripts
Molecular response 4 (MR4)	≤0.01% *BCR::ABL1* transcripts against 10,000 *ABL* reference gene transcripts/24,000 *GUSB* reference gene transcripts
Molecular response 4.5 (MR4.5)	≤0.0032% *BCR::ABL1* transcripts against 32,000 *ABL* reference gene transcripts/77,000 *GUSB* reference gene transcripts

In 1970, Janet Rowley discovered the Philadelphia Chromosome in patients with CML, a shortened chromosome 22 as a result of a translocation with chromosome 9 (t(9;22)(q34;q11)) (Rowley, 1973). We now know this to result in the *BCR::ABL1* fusion oncogene. The breakpoint in the Abelson (*ABL1)* gene is generally constant occurring after exon 2 (a2). In the breakpoint cluster region (*BCR*) gene, however, this is more variable, occurring most commonly after e13 or e14 in CML and e1 and e2 in Ph+ ALL and Ph+ AML. As a result, three chimeric BCR::ABL1 proteins of different molecular weight can be produced (p190, p210 and p230 BCR::ABL1) (Melo, 1997). Rarely, observed variations in breakpoints have been observed (Arber et al., 2016). *BCR::ABL1* encodes a constitutively active oncogenic tyrosine kinase leading to uncontrolled cell proliferation, antiapoptotic effects, arrested lymphoid development and multistep oncogenic progression.

The molecular hallmark expressed in the vast majority of CML cases is the p210 protein, also being expressed in around 25% of Ph+ ALL. The remaining 75% of Ph+ ALL express the p190 BCR::ABL1 protein, with only around 1–2% of CML cases expressing this variant. In addition, 5–7% of CML cases show co-expression of both p190 and p210 isoforms (Molica et al., 2015). The p190 protein lacks the DH–PH domain and when associated with CML is often associated with a monocytosis, absence of splenomegaly and bone marrow morphology which is intermediate between CML and chronic myelomonocytic leukaemia with inferior outcome and short-lived responses to TKIs (Gandhe et al., 2021). The difference between these two isoforms is still poorly characterised, although p190-specific up-regulation of IFN pathways and activation of STAT1 and STAT2 as well as Src and PAK1 kinases has been observed which may explain some of the differences in disease phenotype (Adnan-Awad et al., 2021). The p230 isoform is classically associated with neutrophilic CML (CML-N) demonstrating a lower total white blood cell count, less severe anaemia, less prominent splenomegaly and a more indolent course (Szuber and Tefferi, 2018).

Development of highly efficacious targeted therapeutics in the form of BCR::ABL1-specific TKIs has revolutionised the treatment of CML, leading to a more than 10-fold increase in survival for CML patients without the associated toxicities of alloHSCT. A number of scores including the SOKAL score and EUTOS long-term survival score (ELTS) have been developed to predict response to therapy in CML and are used in many of the studies we will discuss. However, work is still required to validate these scores in the current treatment landscape. ELTS has been demonstrated as a better predictor of outcome, however, further validation is required with the first-line use of second-generation TKI therapies (Pfirrmann et al., 2020).

## Monitoring response to therapy in CML

Monitoring of disease with the use of quantitative reverse transcriptase polymerase chain reaction (qRT-PCR) is paramount to the effective use of TKIs for disease management. *BCR::ABL1* transcripts are compared to a housekeeper gene (usually *ABL1*) allowing for very sensitive assessment of response to TKI therapy with detection of minimal residual disease using the international scale (Table 2), and decisions around switches in therapy to be made early (Hehlmann, 2020; Hochhaus et al., 2020; Smith et al., 2020).

**Table 2.** Treatment milestones in CML

	Optimal response	Warning	Failure
3 months	≤10%	>10%	No CHR
6 months	≤1%	1–10%	>10%
≥12 months	≤0.1%	0.1–1%	>1%

*Note*: *BCR::ABL1* qRT-PCR results expressed on the international scale, highlighting optimal response, warning and treatment failure at specific time points.

## First-generation TKIs

In 1996, preclinical data demonstrated that a modified 2-phenylaminopyrimidine induced apoptosis of BCR::ABL1-positive human cells, including primary CML cells, with little toxicity to normal cells (Druker et al., 1996). This compound, now known as imatinib, specifically inhibited BCR::ABL1 tyrosine kinase activity by competitive inhibition at the ATP-binding site of the BCR::ABL1 protein. This resulted in the inhibition of phosphorylated proteins from downstream signalling pathways. Although imatinib effectively inhibits BCR::ABL1, it also blocks other kinases, including platelet-derived growth factor receptor (PDGFR) and c-KIT (Giles et al., 2009). Its clinical ability was demonstrated in the International Randomised Study of Interferon and STI571 (IRIS) study, where imatinib (STI571) was shown to induce significantly higher rates of haematological and cytogenetic responses, and subsequently dramatically improved overall survival compared to the interferon-alpha plus low-dose cytarabine; the standard-of-care prior to TKIs (O’Brien et al., 2003). In IRIS, initial assessment at a median of 19 months, demonstrated a complete cytogenetic response (CCyR) rate of 74% versus 9% (*p* < 0.001) and failure to progress at 12 months of 99% versus 93% (*p* < 0.001) in the imatinib arm compared to interferon-alpha plus low-dose cytarabine, respectively (O’Brien et al., 2003).

Importantly, this response was durable, with an 8-year follow-up demonstrating event-free survival of 91% and overall survival of 93% (Hochhaus et al., 2009). However, an 8-year follow-up within the trial highlighted that only 55% of patients enrolled remained on imatinib therapy, highlighting potential issues with tolerability and adverse events amongst this patient group (Table 3). In addition, 16% of patients discontinued treatment due to treatment resistance. Although dose escalation of imatinib can improve response rates in patients with a sub-optimal response, switching to second or third-generation TKIs can be more effective (Cortes et al., 2010a, b; Kantarjian et al., 2010). Since the introduction of imatinib, second and third-generation TKIs have been developed.

**Table 3.** Common side effects associated with individual TKIs, degree of severity typically related to effect on other kinase activity affected although in many cases full mechanism of side effects poorly understood

Tyrosine kinase inhibitor	Side effects	Kinase activity affected
Imatinib	Peripheral oedema, GI side effects, muscle cramps, depigmentation	ABL/ARG, cKIT, PDGF-R
Nilotinib	Arterial occlusion, hyperglycaemia, pancreatitis	ABL/ARG, cKIT, PDGF-R
Dasatinib	Pleural effusion, cytopenias, pulmonary hypertension (rare)	ABL/ARG, Src, cKIT, PDGF-R
Bosutinib	GI side effects, pleural effusions, elevated transaminases	Src, ABL/ARG
Ponatinib	Hypertension, venous and arterial thromboembolism, cardiovascular disease	ABL/ARG, Src, cKIT, PDGF-R, VEGF-R

*Note*: Under physiological circumstances, ABL/ARG regulates response to DNA damage and oxidative stress; Src kinases involved with haematopoiesis, innate and adaptive immune response and vascular permeability; c-KIT regulates haematopoiesis, GI motility and melanogenesis; PDGF-R regulation of interstitial fluid pressures, VEGF-R cardiac homeostasis and angiogenesis.

## Second- and third-generation TKIs

Dasatinib, nilotinib, bosutinib and ponatinib have led to further improvement in CML outcome. Dasatinib is a dual SRC/ABL1 second-generation TKI shown to be significantly more potent when compared to imatinib *in vitro.* The DASISION trial (NCT00481247) compared dasatinib 100 mg OD and imatinib 400 mg OD in the setting of front-line treatment (Kantarjian et al., 2010). At 12 months, the primary outcome of confirmed CCyR was 83% versus 72% (*p* = 0.007) in the dasatinib and imatinib arms, respectively (Jabbour and Kantarjian, 2014). Five-year follow-up demonstrated that dasatinib achieved deeper responses at earlier time points, with a higher proportion of patients achieving major molecular response (MMR) at 3 months in the dasatinib arm (84% versus 64%, *p* < 0.0001) (Cortes et al., 2016). There was also less frequent progression to BP-CML. Rates of grade 3 or 4 haematologic adverse events were higher for dasatinib versus imatinib. The UK STI571 Prospective International Randomised Trial 2 (SPIRIT2) phase 3 clinical trial confirmed these results, with 12-month MMR being achieved in 57.5% versus 46% in the dasatinib and imatinib arms, respectively (*p* < 0.001) (Osborne et al., 2015). Again, significant treatment toxicities were evident with dasatinib treatment, with pleural effusions occurring more frequently with dasatinib than with imatinib (24.1% versus 1.2%) (Table 3) (Osborne et al., 2015). In patients intolerant or resistant to imatinib, dasatinib has demonstrated durable response rates, with the START-C phase 2 study demonstrating a 2-year CCyR rate of 53%, with 90% of these being maintained at 24 months. Furthermore, the START-R study demonstrated significantly higher rates of CCyR comparing dasatinib 70 mg twice daily with high-dose imatinib at 2 years (i.e., 400 mg twice daily) (44% versus 18%, *p* = 0.0025) (Kantarjian et al., 2009; Milojkovic et al., 2012).

Nilotinib, a structural analogue to imatinib, has increased affinity for BCR::ABL1 *in vitro* compared to imatinib (Weisberg et al., 2005). Like dasatinib, nilotinib has been shown to induce a sustained haematological and cytogenetic response in more patients, when compared with imatinib. The Evaluating Nilotinib Efficacy and Safety in Clinical Trials – Newly Diagnosed patients (ENESTnd) study was a phase 3, randomised clinical trial comparing two doses of nilotinib (300 mg BD and 400 mg BD) to imatinib (400 mg OD). The use of nilotinib was associated with significantly higher MMR at 12 months compared to imatinib, with limited change between the two doses of nilotinib used (44% versus 43% versus 22% for nilotinib 300 mg BD, nilotinib 400 mg BD and imatinib 400 mg OD, respectively; *p* < 0.001) (Saglio et al., 2010; Kantarjian et al., 2011). Five-year follow-up demonstrated the cumulative incidence of MMR to be 77% for both doses of nilotinib and 60% with imatinib (*p* < 0.0001) (Saglio et al., 2010; Kantarjian et al., 2011). Although event-free survival was not statistically changed between trial arms, there was an advantage with reducing rates of disease progression in those with intermediate and high-risk disease receiving nilotinib, as estimated through the Sokal score. This highlights the role for second-generation TKIs in prevention of disease transformation. As with dasatinib, adverse events and toxicity with nilotinib use are significant. Within the ENESTnd study, the 6-year cumulative cardiovascular side effect event (CVE) rate was significantly increased in the nilotinib arm, with a dose-dependent effect (Hochhaus et al., 2016). CVEs were reported in 46 (16.5%), 65 (23.5%) and 10 (3.6%) patients, respectively, in the nilotinib 300-mg twice daily, nilotinib 400-mg twice daily and imatinib arms. Sub-analysis demonstrated higher baseline Framingham general cardiovascular risk scores were predictive of patients’ risk of developing a CVE during treatment (Table 3) (Kantarjian et al., 2021).

Bosutinib is a dual SRC/ABL TKI that demonstrates more potent *in vitro* activity against BCR::ABL1 than imatinib but has less activity against c-KIT and PDGFR (Puttini et al., 2006). The phase 3 clinical trial, bosutinib efficacy and safety in newly diagnosed chronic myeloid leukaemia (BELA), compared the efficacy and safety of bosutinib against imatinib (Cortes et al., 2012). Following a dose escalation to 500 mg OD, the trial allowed the potential for a further increase to 600 mg OD for patients with inadequate molecular and cytogenetic response. At 24 months, cumulative CCyR was similar in bosutinib (79%) and imatinib (80%) arms; however, cumulative MMR rates were significantly higher with bosutinib (59% versus 49%) (Brümmendorf et al., 2015). Importantly, bosutinib appeared to retain activity across mutations that confer imatinib resistance with the exception of T315I and responses were independent of whether patients had resistance or intolerance to imatinib (Nakaseko et al., 2015). These results were also supported by the BFORE study which looked at 536 patients with newly diagnosed CP-CML assigned to receive 400 mg of bosutinib once daily (*n* = 268) or imatinib (*n* = 268). Achievement of MMR at 12 months was 47.2% versus 36.9%, respectively (*p*= 0.02) (Cortes et al., 2018).

Ponatinib is a multi-targeted kinase inhibitor and is currently the only approved TKI active against the T315I BCR-ABL kinase domain mutation (see below; O’Hare et al., 2009), and therefore is an important treatment modality in BCR::ABL1-positive leukaemias. The Ponatinib Ph-positive ALL and CML Evaluation (PACE) trial evaluated 449 patients that were considered resistant or intolerant of second-generation TKIs or harboured T315I mutations (Cortes et al., 2013). The trial used a dose of 45 mg OD ponatinib and patients were stratified by both disease phase and mutational status. At 12 months, major cytogenetic response (MCyR) was achieved in 56% of the patient cohort, with 75% of patients harbouring the T315I mutation achieving MCyR. Long-term follow-up confirmed a sustained response, with 83% of these patients remaining in MCyR at 3 years and 39% of patients achieving MMR or better (Cortes et al., 2014). As with other TKIs, significant adverse events were noted. Particularly, arterial occlusive events occurred in 28% of patients (Cortes et al., 2014). Through its multi-kinase inhibitory properties, including inhibition of vascular endothelial growth factor (VEGF), it has been postulated ponatinib causes endothelial dysfunction and hypertension, and can promote proatherogenic surface adhesion receptors thus increasing the risk of vascular occlusive events (Table 3; Chan et al., 2020). The OPTIC trial looked at ponatinib dose ranging to identify optimal benefit/risk outcomes; 45 mg, 30 mg and 15 mg starting doses were used with those in the 45 mg and 30 mg cohort being required to have their dose reduced to 15 mg once daily upon achievement of ≤1% *BCR::ABL1.* Rates of ≤1% *BCR::ABL1* at 12 months were 44.1%, 29.0% and 23.1%, respectively, with subgroup analysis demonstrating poorer response in those with a T315I mutation. However, in those achieving response, this was maintained with dose reduction in 73.3% and 78.6% of patients in the 45 mg and 30 mg cohorts, respectively; with most of those losing response demonstrating the T315I mutation at baseline. The trial concluded that to maximise response while minimising toxicity a 45 mg starting dose reduced to 15 mg on achieving BCR::ABL1 ≤ 1% is recommended (Cortes et al., 2021).

## TKI resistance – BCR-AB1-dependent and independent mechanisms

### BCR-ABL-dependent mechanisms

Point mutations or amplification at the kinase domain of the *BCR::ABL1* protein are the most common mechanism by which cells develop resistance to TKIs. More than 90 different BCR::ABL1 kinase domain point mutations have been identified, with a lesser number biologically characterised (Azevedo et al., 2017).

BCR::ABL kinase domain mutations include those that affect the P-loop, SH2 domain, SH3 domain and the activation loop (Branford et al., 2003; Soverini et al., 2006). Mutation type can be associated with phase of disease; M244, M351 and G250 are frequently detected in CP, and mutations at T315, E255 and Y253 are typically found in BP (Soverini et al., 2006; Branford et al., 2009). The T315I mutation, resulting in the replacement of threonine by isoleucine at the ABL amino acid position 315, is of particular clinical relevance as ponatinib remains the only therapeutic option. It is also important to note that not all mutations result in TKI resistance. Identification of specific mutations is therefore imperative to guide TKI choice (Hehlmann, 2020; Smith et al., 2020).

The ELN recommends screening for mutations in any patient with treatment failure or warning response, including a rise in *BCR::ABL1* transcript levels, to allow for early detection of mutations and appropriate change in therapy (Hehlmann, 2020).

### BCR-ABL-independent mechanisms

BCR-ABL1-independent mechanisms of resistance are also described, however, remain poorly understood. These typically result from alterations of cellular signalling and cell cycle regulation downstream of the BCR::ABL1 protein (Li and Li, 2007). BCR::ABL1 activates alternative signalling pathways, including SRC kinases, RAS and JAK–STAT (Gallipoli et al., 2014). Aberrant activation of these pathways leads to the CML cell’s proliferation and enhanced survival. The activation of the SRC family kinases has been shown to promote both disease progression, as well as TKI-unresponsiveness. BCR::ABL1 directly interacts with SRC family kinases resulting in both a conformational change in the ABL kinase domain through phosphorylation of SH2 and SH3, as well as activation of SRC family kinases, Hck, Lyn and Fyn, leading to cell growth, differentiation and survival (Stanglmaier et al., 2003; Hu et al., 2006; Meyn et al., 2006). Additionally, while there have been no clinical examples of SRC-activating mutations in imatinib-resistant cell lines or primary CML specimens, cellular activation of this pathway through numerous other crosstalk networks may still facilitate resistance (Donato et al., 2003; Ptasznik et al., 2004; Wu et al., 2008). RAS signalling is also activated through Grb2-mediated binding of the Y177 moiety in the BCR sequence, resulting in mitogen-activated protein kinases activation (Cortez et al., 1997).

Up-regulation of intracellular efflux transporters and down-regulation of influx transporters has also been demonstrated to confer resistance (Sattler et al., 2002). Grb2 can recruit Gab-2 with subsequent activation of both PI3k and ERK pathways (Sattler et al., 2002). The mechanism by which these signalling pathways are activated has been shown to be through the expression of plasma membrane transporter molecules, including the ABC family of transporters, ABCG2 and MDR-1. It has been suggested that over-expression of these protein complexes might confer resistance through reducing intracellular imatinib concentration (Mahon et al., 2003). However, a clear correlation between transporter molecule expression and patient response to imatinib has not been fully elucidated (Mahon et al., 2003).

More recently, greater understanding of the leukaemic stem cell (LSC) and its role in TKI resistance has become apparent. LSCs have been shown to utilise multiple cell-intrinsic pathways, together with microenvironmental and immune cell interactions to evade current therapies including TKIs and have been linked to treatment-refractoriness and disease progression (Hsieh et al., 2021). Mitochondrial oxidative phosphorylation within CML LSCs has been well described. Mitochondrial reactive oxygen species are increased in quiescent CML LSCs resulting in increased oxidative DNA damage, genetic instability and clonal evolution (Nieborowska-Skorska et al., 2012). Additionally, dysregulation of apoptosis through up-regulation of the BH3 family of proteins including the antiapoptotic proteins MCL-1, BCLxL and BCL2 leads to persistence of LSCs despite treatment (Vetrie et al., 2020). Carter and colleagues have recently demonstrated eradication of the CD34+ quiescent progenitor cells from BP-CML patient samples using the BCL2 inhibitor, venetoclax, in combination with TKIs (Carter et al., 2016). Furthermore, clinical trials evaluating BCL2 inhibitors in combination with TKI are currently underway (Jabbour and Kantarjian, 2020).

Epigenetic methylation and post-translation acetylation alter gene expression and subsequent protein function. Within CML, the role of epigenetic modification at a biological and therapeutic level are becoming of increasing interest (Scott et al., 2016). Pre-leukaemic mutations in epigenetic regulators (DNMT3A, TET1, TET2, IDH1 and IDH2) provide the most obvious underlying basis for epigenetic re-patterning in CML (Jan et al., 2012; Shlush et al., 2014). These changes can lead to functional changes with up-regulation of mitochondrial metabolism and antiapoptotic effects leading to persistence of LSCs despite optimal treatment (Holyoake and Vetrie, 2017; Vetrie et al., 2020).

## STAMP inhibitors

Asciminib is the most recent drug to be approved for the treatment of CML and is a breakthrough for the small proportion of patients whose disease fails to respond to two or more TKIs. Asciminib is a first in class BCR::ABL1 STAMP inhibitor. Wild-type ABL has a myristoylated N-terminus which is not present in the BCR::ABL1 fusion oncoprotein. Under normal circumstances binding of the terminus to an allosteric site leads to reduced activity, however, when absent as in the BCR::ABL1 fusion protein, this regulation is lost and the protein is constitutively active. Asciminib binds to the allosteric site inhibiting protein activity (Schoepfer et al., 2018). Very recently, the phase 3 ASCEMBL clinical trial compared asciminib 40 mg twice daily with bosutinib 500 mg once daily in patients that had failed two or more TKIs, and demonstrated MMR of 25.5% and 12.2%, respectively (*p* = 0.029). There were also fewer discontinuations with asciminib, 5.8% compared to 21.1% (Réa et al., 2021). Asciminib is now approved for third or later line use in patients with TKI resistance or intolerance.

## Choosing the right therapy

First-line therapy for CP-CML is invariably TKI therapy, with imatinib, dasatinib, nilotinib or bosutinib all approved for this indication. Generic imatinib is now deemed the most cost-effective first-line therapy in most patients. Although faster responses are seen with the second-generation TKIs, randomised trials with 5- and 10-year follow-up demonstrated no survival benefit (Cortes et al., 2016; Hochhaus et al., 2016). Given the ever-expanding data, it is important to consider patient comorbidities and BCR::ABL1 mutation status when deciding on TKI therapy (Table 4).

**Table 4.** Decisions on most appropriate therapy, based on BCR::ABL1 kinase domain mutations, relative contra-indications, and current FDA approvals for the different TKIs

TKI	BCR::ABL1 kinase domain resistance mutations which can be overcome	Relative contraindications	Starting dose	Current FDA approval
Imatinib	N/A	None	400 mg/daily in CP 400 mg twice daily in BP-CML	1st line CP and BP-CML or Ph+ ALL
Dasatinib	Y253H, E255V/K, F359V/I/C	Prior pleural or lung disease	100 mg/daily in CP and 140 mg/daily in BP-CML	1st line/2nd line CP and BP-CML or Ph+ ALL
Nilotinib	F317L/VLI/C, T315A, V299L	History of coronary artery disease or cerebrovascular disease	1st line – 300 mg/Twice daily 2nd line – 400 mg/twice daily	1st line/2nd line CP-CML
Bosutinib	Y253F/H, E279K, M351T, H396P/R	Abnormal liver function tests	1st line – 400 mg/daily 2nd line – 500 mg/daily	1st line/2nd and later lines in CP or BP-CML
Ponatinib	Almost all BCR::ABL1 KD mutations, including T315I	Coronary artery disease, peripheral vascular disease	45 mg/daily reducing to 15 mg daily on achieving CCyR	2nd line if T315I mutation otherwise 3rd line if treatment failure with nilotinib or dasatinib in CP or BP-CML, or Ph+ ALL
Asciminib	All BCR::ABL1 KD mutations, including T315I	None	40 mg/twice daily	3rd line CP-CML

For second-line therapy, where there is intolerance or resistance to first-line TKI, confirmation of compliance is essential in the first instance. Mutational analysis with next-generation sequencing should be performed, with mutations being identified in 4.6% of CP-CML patients and in up to 80% of patients with BP-CML (Branford et al., 2018). Consideration of mutation analysis and patient characteristics, including co-morbidities, should then be made to provide individualised treatment decisions. Ponatinib remains reserved for those patients where a T315I mutation is present or where there has been failure of two previous lines of therapy (Hehlmann, 2020; Copland, 2022; Copland et al., 2022).

Despite the significant improvements in outcomes for CP-CML with the advancement in TKI therapy, outcomes for patients presenting in BP or developing BP-CML while receiving TKI therapy remain dismal with alloHSCT being the only treatment option offering the chance of long-term remission or cure. Clonal evolution with acquisition of further oncogenic mutations leads to lymphoid, myeloid or biphenotypic acute leukaemias. Treatment depends primarily on whether BP-CML has developed on treatment. For those fit for alloHSCT without prior TKI exposure, *de novo* BP treatment is typically with 800 mg imatinib or 140 mg dasatinib, alongside AML or ALL standard chemotherapy approaches, to achieve cytogenetic remission and return to CP before proceeding to HSCT. In those progressing to BP on TKI, the approach is similar, however, ponatinib may be more attractive here. This approach has been supported in the recently published phase 1/2 MATCHPOINT trial which used either 1 or 2 cycles of fludarabine, cytarabine, granulocyte-colony stimulating factor and idarubicin (FLAG-IDA) chemotherapy in combination with ponatinib 30 mg daily before proceeding to alloHSCT with median OS of 12 months and 41% of patients being alive at 3 years (Copland, 2022; Copland et al., 2022).

## Dose optimisation and treatment-free remission

Widespread use of TKIs means we now have a plenitude of “real-world” and clinical trial data highlighting the safety of dose modification in maintaining adequate treatment response, while optimising compliance and reducing complications. Prospective clinical trials are still required to aid further improvement to see if we are over-treating optimally responding patients and to support current evidence of dose modifications in patients with intolerable side-effect profiles.

Prospective clinical trials have explored the safety of TKI discontinuation in CP patients with sustained deep molecular remission (DMR; MR4 or better) (Mahon et al., 2010; Mahon, 2015; Etienne et al., 2017). The Stop Imatinib’ (STIM) trial demonstrated an incidence of molecular recurrence at 60 months post cessation of imatinib of 61% (CI 52–70%), with few cases of late recurrence being observed (Mahon et al., 2014). This suggests that if molecular recurrence is to occur, it happens early. These data are supported by the European Stop Kinase Inhibitor (EURO-SKI) trial (Mahon et al., 2014, 2016, 2021), which recruited 868 patients of whom 728 were eligible for analysis. Median time on TKI treatment was 7.5 years (range 3.0–14.1 years) with median duration in MR4 before cessation of 4.7 years. 46% of the patients, were still in MMR at 3 years demonstrating cessation of treatment is safe in a significant minority of patients, however, unlike in STIM, late molecular recurrence was more prevalent at 15% between 6 and 36 months emphasising the need for ongoing long-term close monitoring in these patients (Mahon et al., 2021). Importantly those patients with molecular recurrence will rapidly re-achieve deep molecular responses on re-starting TKI therapy.

The DESTINY study (Clark et al., 2019) looked at the role of dose reduction prior to treatment cessation of imatinib, nilotinib or dasatinib in CML and demonstrated that reducing TKI dose to half standard treatment dose was associated with improved tolerability without negative impact on disease control in those with stable MR3 or better. TKI dose was reduced for 12 months prior to cessation of treatment. The trial looked at 49 patients in MMR and 125 in the DMR at the time of dose reduction. In the DMR group, 84 (67%) patients reached the 36-month trial completion point and recurrence-free survival was 72% (95% CI 64–80). In the MMR group, 16 (33%) entrants completed the study and recurrence-free survival was 36% (95% CI 25–53) (Clark et al., 2019). The higher rates of ongoing DMR in the DESTINY study raise the question of whether initial dose reduction prior to complete cessation of treatment improves outcome. Potential mechanisms are unclear and raise questions around the potential trigger events leading to molecular recurrence. The role of dose reduction on LSC persistence, the bone marrow microenvironment and compliance may all be implicated and requires further work to understand. It does support the argument that treatment cessation for the time being should be reserved for those patients with sustained DMR.

## Role in acute leukaemias

The Ph chromosome is the most common cytogenetic abnormality in adults with ALL, being associated with 20–30% of cases. It was historically associated with a poor prognosis, with an increased risk of CNS involvement and an aggressive clinical course. TKIs are now routinely incorporated into standard treatment protocols and have improved outcomes, with initial trials demonstrating a complete response (CR) rate of 92% versus 82% with the addition of imatinib to treatment (Fielding et al., 2014). However, relapse rates remain high and alloHSCT in first cytogenetic remission for those suitable remains key for long-term survival (Liu-Dumlao et al., 2012). Paediatric studies have raised the possibility of omitting alloHSCT in the first CR, however, as yet there is insufficient data to incorporate this into adult practice (Ravandi, 2019). The increasing use of anti-CD19 and anti-CD22 immunotherapy in the form of blinatumomab and inotuzumab ozogamicin, respectively, as well as chimeric antigen receptor T cell therapy (CAR-T) for ALL is likely to change the landscape of managing Ph+ ALL in the coming years. The phase 2 GIMEMA D-ALBA trial looked at the use of blinatumomab in combination with dasatinib with omission of standard cytotoxic chemotherapy as upfront treatment for Ph+ ALL and demonstrated 3-year overall survival of 80% and disease-free survival of 71%. Further work and longer follow up is required, however, this is the first suggestion that it may be possible to omit cytotoxic chemotherapy from Ph+ ALL treatment (Foà et al., 2020; Curran et al., 2022).

The use of prophylactic TKI therapy to prevent relapse post-alloHSCT has been investigated, however, there remains no firm evidence on which patients will benefit. Guidelines currently recommend close monitoring of marrow and peripheral blood for *BCR::ABL1* MRD. All patients should be offered prophylactic treatment for a minimum of 12 months with negative MRD if alloHSCT is performed in CR1 and switched to ponatinib in the event of *BCR::ABL1* re-appearance (Ravandi, 2019).

The choice of TKI remains an area of discussion. Dasatinib has been shown to have increased CNS penetration compared to imatinib, and as such, it has been hypothesised that its use may reduce CNS relapse in particular (Shen et al., 2020). Shen et al. published a randomised clinical trial of imatinib versus dasatinib for paediatric patients demonstrating relapse rates of 34.4% and 19.8% (*p* = 0.01) with CNS relapse rates of 8.4% and 2.7% (*p* = 0.06), respectively (Shen et al., 2020). The European Society for Blood and Marrow Transplantation (EBMT) recommends dasatinib for patients with a prior history of CNS disease. Further research is required to confirm this in a European adult population.

Acute myeloid leukaemia with BCR::ABL1 fusion is a defined genetic entity in the fifth World Health Organization classification (Khoury et al., 2022). Given the rarity of this condition there is little evidence on the use of TKIs, however, a number of case reports have demonstrated improved outcomes when incorporated into standard treatment. Ph+ AML, even with the advent of TKIs, has a poor prognosis and should be treated with consolidative alloHSCT if CR is reached and this is a viable treatment option (Mizuno et al., 2021).

## Conclusion

Our knowledge of BCR::ABL1-positive leukaemias continues to evolve and with this, a personalised approach to leukaemia management continues to expand. The discovery of TKIs has revolutionised outcomes for patients and provides a key example of 21st century precision medicine. CML management should now be considered as patient-specific, considering patient co-morbidities and lifestyle. For patients with intermediate or high-risk disease, as dictated through the prognostic scoring systems, there should be strong consideration for a second-generation TKIs upfront (Ciftciler and Haznedaroglu, 2021).

There remain many unanswered questions and ongoing clinical trials exploring BCL2 inhibitors as well as other agents to specifically target quiescent stem cell populations, and increasing use of STAMP inhibitors for refractory cases are likely to improve outcomes in CML (Jabbour and Kantarjian, 2020). Additionally, immunotherapy and CAR-T in Ph+ ALL are expanding areas. Critical areas of unmet need remain, including management of patients with TKI resistance and BP-CML, increasing the number of patients able to attempt and maintain successful treatment-free remission, and improvements in dose optimization and TKI selection to minimise side effects of long-term therapy with further research required.
