# Supplementary material for: Targeting BCR-ABL1-positive leukaemias: a review article
Source: Camb Prism Precis Med. 2023 Mar 6;1:e21. doi: 10.1017/pcm.2023.9 (PMC10953774; doi:10.1017/pcm.2023.9)
Supplement: Peer review history

# Reviewer report: Review: Targeting BCR-ABL1-positive leukaemias, a review article — R0/PR2

**DOI:** 10.1017/pcm.2023.9.pr2

**Authors:** Ibrahim C. Haznedaroglu

**Affiliations:** Hacettepe University

*Comments to Author*: The manuscript describes important novel data in the studied area and worth to be published with some improvements in the presentation style and language. Discussion section shall be expanded via additional perspectives regarding previous papers in the CML field (such as; Tailored tyrosine kinase inhibitor (TKI) treatment of chronic myeloid leukemia (CML) based on current evidence. by Ciftciler R, et al. Eur Rev Med Pharmacol Sci. 2021 Dec;25(24):7787-7798. doi: 10.26355/eurrev_202112_27625.)

# Editor report: Recommendation: Targeting BCR-ABL1-positive leukaemias, a review article — R0/PR3

**DOI:** 10.1017/pcm.2023.9.pr3

**Affiliations:** Institute for Molecular Medicine Finland, University of Helsinki, Finland

*Comments to Author*: Please consider expanding the concluding remarks as suggested by the reviewer. Also, the manuscript should be reviewed to correct minor typographical errors.

# Editor report: Decision: Targeting BCR-ABL1-positive leukaemias, a review article — R0/PR4

**DOI:** 10.1017/pcm.2023.9.pr4

**Affiliations:** Institute of Cardiovascular & Medical Sciences, University of Glasgow College of Medical Veterinary and Life Sciences, United Kingdom of Great Britain and Northern Ireland

No accompanying comment.

# Reviewer report: Review: Targeting BCR-ABL1-positive leukaemias, a review article — R1/PR6

**DOI:** 10.1017/pcm.2023.9.pr6

**Authors:** Ibrahim C. Haznedaroglu

**Affiliations:** Hacettepe University

*Comments to Author*: The paper may be accepted in its present form.

# Editor report: Recommendation: Targeting BCR-ABL1-positive leukaemias, a review article — R1/PR7

**DOI:** 10.1017/pcm.2023.9.pr7

**Affiliations:** Institute for Molecular Medicine Finland, University of Helsinki, Finland

No accompanying comment.

# Editor report: Decision: Targeting BCR-ABL1-positive leukaemias, a review article — R1/PR8

**DOI:** 10.1017/pcm.2023.9.pr8

**Affiliations:** Institute of Cardiovascular & Medical Sciences, University of Glasgow College of Medical Veterinary and Life Sciences, United Kingdom of Great Britain and Northern Ireland

No accompanying comment.
